# AN UNEQUAL WORK ENVIRONMENT: NURSING HOME STAFF PERCEPTIONS OF UNIT ORGANIZATIONAL CONTEXT

**DOI:** 10.1093/geroni/igad104.2847

**Published:** 2023-12-21

**Authors:** Alba Iaconi, Kaitlyn Tate, Tatiana Penconek, Yinfei Duan, Seyedehtanaz Saeidzadeh, Greta Cummings, Peter Norton, Carole Estabrooks

**Affiliations:** University of Alberta, Edmonton, Alberta, Canada; University of Alberta, Edmonton, Alberta, Canada; University of Alberta, Edmonton, Alberta, Canada; University of Alberta, Edmonton, Alberta, Canada; University of Alberta, Edmonton, Alberta, Canada; University of Alberta, Edmonton, Alberta, Canada; University of Calgary, Calgary, Alberta, Canada; University of Alberta, Edmonton, Alberta, Canada

## Abstract

The organizational context in nursing homes is associated with quality of care and residents’ quality of life. Our objective was to examine nursing home staff perceptions of unit organizational context. We conducted a secondary analysis of cross-sectional data. Participants were 3765 unregulated (health care aides (HCAs)) and 1130 regulated staff (managers, registered nurses, registered psychiatric nurses (RN/RPNs) and licensed practical nurses (LPNs)) from 91 nursing homes in Western Canada. We measured nursing home staff’s perceptions of organizational context via the Alberta Context Tool (ACT), comprised of ten concepts: leadership, culture, evaluation, social capital, formal interactions, informal interactions, organizational slack (staff, space, time). Differences between groups were examined via one-way ANOVA. Managers reported significantly higher organizational context scores compared to all other staff members in care homes, followed by RN/RPNs. LPNs and HCAs reported the lowest scores. Specifically, LPNs reported the lowest scores on all organizational context measures except for structural resources and organizational slack space, for which HCAs reported the lowest scores. HCAs reported higher scores than LPNs on unit leadership, culture, evaluation, social capital. When compared with managers and RN/RPNs in the nursing home, LPNs reported the lowest SF-8 mental health scores (49.30 (10.05)) and HCAs reported the lowest physical health scores (48.53 (8.16)). Our work demonstrates the need for improvement of staffing and time for staff in nursing homes. Specific attention is needed to focus on modifiable organizational unit context factors for LPNs and HCAs, who are responsible for providing the majority of the direct care for residents.

